# LiDAR-Based Structural Health Monitoring: Applications in Civil Infrastructure Systems

**DOI:** 10.3390/s22124610

**Published:** 2022-06-18

**Authors:** Elise Kaartinen, Kyle Dunphy, Ayan Sadhu

**Affiliations:** Department of Civil and Environmental Engineering, Western University, London, ON N6A 3K7, Canada; ekaartin@uwo.ca (E.K.); kdunphy2@uwo.ca (K.D.)

**Keywords:** terrestrial laser scanning, mobile laser scanning, structural assessment, automation, damage detection, quality control

## Abstract

As innovative technologies emerge, extensive research has been undertaken to develop new structural health monitoring procedures. The current methods, involving on-site visual inspections, have proven to be costly, time-consuming, labor-intensive, and highly subjective for assessing the safety and integrity of civil infrastructures. Mobile and stationary LiDAR (Light Detection and Ranging) devices have significant potential for damage detection, as the scans provide detailed geometric information about the structures being evaluated. This paper reviews the recent developments for LiDAR-based structural health monitoring, in particular, for detecting cracks, deformation, defects, or changes to structures over time. In this regard, mobile laser scanning (MLS) and terrestrial laser scanning (TLS), specific to structural health monitoring, were reviewed for a wide range of civil infrastructure systems, including bridges, roads and pavements, tunnels and arch structures, post-disaster reconnaissance, historical and heritage structures, roofs, and retaining walls. Finally, the existing limitations and future research directions of LiDAR technology for structural health monitoring are discussed in detail.

## 1. Introduction

Infrastructure, including roads, bridges, and buildings, has significantly deteriorated on a global scale, as many of these structures have a limited duration of exploitation. The prevalence of deteriorating structures, in combination with the increased frequency of natural disaster events within recent decades, has necessitated the development of inspection techniques to ascertain the damage and overall level of safety of structures. Often the nature of these structural damages results in logistic constraints, by which human-based visual inspections cannot be conducted, as they pose a danger to the inspectors’ health or the area under inspection cannot be accessed by normal means. Additionally, the use of contact-based devices is time-consuming as they require an extensive setup to attach the devices to the structure and initiate the data collection process. This ultimately delays the rehabilitation process for damaged structures, often resulting in economic losses. Therefore, the need for rapid inspection methods which implement non-contact sensors within the field of Structural Health Monitoring (SHM) has become more prevalent within the past decades. 

A non-contact sensor [1] can be defined as any sensor which is implemented for monitoring and data acquisition that does not require the sensor to have physical contact with the structures. Broadly, these devices can be classified into two categories of data collection techniques, based on the nature of data collection: (1) passive and (2) active. Passive techniques use optical setup to take images or videos of the damaged structures, which can later be analyzed by specialists to determine the level of damage present and the necessary rehabilitation technique required. Active techniques emit various spectral bands and frequencies of light and sound which are directed at the structure. The device then measures the Doppler shifting of the returning sound wave or reflected light to ascertain the nature of anomalies in the object under investigation. They address the limitations of human-based inspections as they allow for inspections to be conducted without physical intervention, and their generally efficient setup allows for inspections to be conducted quickly.

The growing popularity of non-contact sensors with SHM has resulted in cameras, smartphones, unmanned aerial vehicles, satellites, ultrasonic devices, and various other sensors being used to conduct structural inspections [1]. Optical-based sensors, such as cameras, smartphones, unmanned aerial vehicles, and satellites, capture an image or video-based data, which can be used to assess the overall stability of a structure. The data is often directly stored on the device that captures the image-based information or can be transmitted back to a base station for further analysis in the case of mobile optical sensors. The quality of data extracted from these devices is highly susceptible to environmental factors, such as lighting, wind, and mechanical vibrations due to moving machine parts [1,2]. Moreover, markers must often be affixed to the structure to quantify any physical change in the structure during monitoring, including displacement or strain. Alternatively, acoustic-based sensors, such as ultrasonic devices, detect damages in structural elements, such as cracks and delamination, through the variation of ultrasonic waves as they propagate through the structural mass. However, non-contact ultrasonic devices are limited to analyzing the surface of the structure, which only provides details on surficial damages. Moreover, these types of devices have numerous variants which have limited applications to a specific type of monitoring, making it difficult to implement in a generalized setting [2].

Light Detection and Ranging (LiDAR) devices and methods have received significant consideration in the past decade by the SHM research community as a tool for 3D point cloud generation and analysis of structures. Recently, many studies have proposed methodologies to optimize the collection and processing of data to create more time-efficient, and computationally inexpensive, SHM systems. An unmanned aerial vehicle-based LiDAR with an optimized flight path decision-making paradigm was implemented by [3] for the inspection of surface defects for bridges. Furthermore, state-of-the-art algorithms have been proposed for the detection of structural elements and damages within millimeters of precision, while minimizing error [4,5]. Over the past decade, several literature reviews have been conducted by researchers pertaining to the implementation of LiDAR devices for structural health monitoring. However, the majority of published literature review papers broadly assess non-contact sensing techniques for damage detection, rather than focusing on LiDAR devices, specifically [6,7,8,9,10,11,12]. Furthermore, current LiDAR-based literature review papers focus on very limited domains within SHM, including deformation monitoring of buildings [13], bridge inspection [14,15], and moisture detection in structures [16]. Therefore, the objectives of the proposed review summarized in this paper are as follows:

1. Provide a comprehensive review of LiDAR-based SHM assessment for various aging civil infrastructure systems, including bridges, tunnels, roads, roofs, walls, buildings, and historical structures.
2. Summarize the existing limitations and gap areas for LiDAR-based SHM technology.
3. Articulate the future research direction of the LiDAR-based monitoring and inspection techniques for SHM.

## 2. Background of LiDAR

Applications of LiDAR for SHM typically involve analyzing 3D objects, structural geometries, deformations, crack information, and visualization using a dense 3D dataset with finer resolution and precision [6,7,8,9,10,11,12,13,14,15,16]. In general, the applications are of two types: (a) phase-based (rapid but limited to short distances) and (b) time-of-flight-based (able to measure large distances, but slow and less accurate). Various laser scanners are currently available with a range of speeds, typically 2000–120,000 points per second, maximum resolutions typically 1–100 mm at 50 m, and accuracies typically 3–50 mm at 100 m [10,11,12,13,17,18,19]. They can be used for as-built building information modeling, structural inspection, and reconnaissance surveys, by capturing 3D data and meta-data of the target several hundred meters away to a precision of a few millimeters. 

### 2.1. Advantages and Limitations of LiDAR Sensors

A potential advantage of LiDAR devices over traditional non-contact sensors is that there is no requirement for physical instrumentation and accessibility of the structures, thereby saving a significant amount of time and labor costs and improving the safety of the workplace. Moreover, these devices are independent of natural light sources, serving as excellent field measurement tools. However, LiDAR has several following limitations that prompted SHM researchers to advance this technology over the last several years.

1. Dependency on weather conditions.
2. Expensive, and performance degrades with distance.
3. Non-structural components must often be removed using specific filters.
4. The datasets captured by LiDAR devices are extremely large, meaning that the computational processing time is long.
5. Strategic positioning is required to avoid overlapping or targetless scans. The field of view, or the angle covered by the sensor, impacts what is captured by the sensor.
6. There is often the need for multiple scans at different points; thus, object extraction or interpolation techniques are required.

### 2.2. Types of LiDAR Sensors

Depending on the mounting mechanism, their application can be categorized into two classes, based on mode of operation: (a) stationary and (b) mobile.

#### 2.2.1. TLS

**Figure 1:**
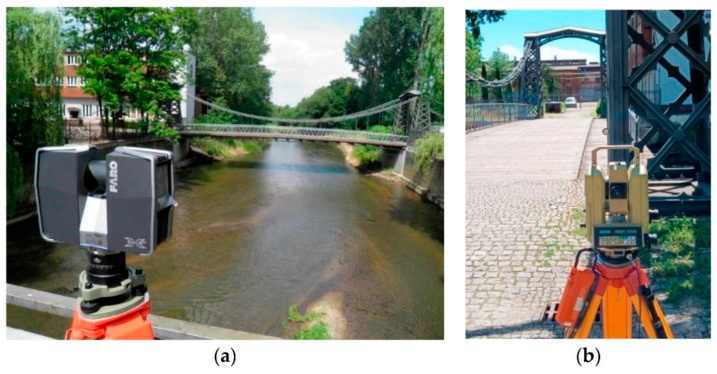
A typical TLS: (**a**) FARO Focus 130 3D laser scanner; (**b**) Leica TC2002 total station [20].

Terrestrial laser scanning (TLS) is a stationary instrument (as shown in Figure 1) that can acquire dense point clouds, often taken from various locations close to the ground. For SHM, TLS scans are captured and merged to create larger point clouds. The size of this point cloud depends on whether the scanner is short-range, medium-range, or long-range. Although using TLS often requires more manual labor and time, these point clouds are generally more detailed and precise than mobile laser scanning (MLS). 

#### 2.2.2. ALS

**Figure 2:**
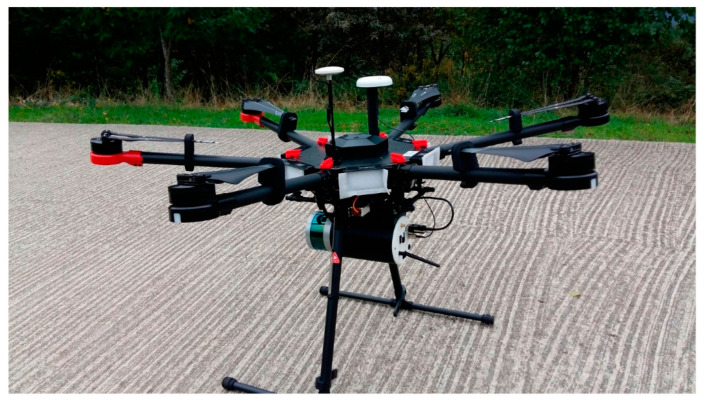
A typical ALS integrated into a drone [21].

Mobile Laser Scanning (MLS) refers to a broad category of devices/instruments that are mounted on mobile equipment, such as drones, airplanes, cars, or helicopters, as shown in Figure 2. Airborne Laser Scanning (ALS) is particularly useful for acquiring point cloud data of large areas of land (e.g., towns, neighborhoods, or cities), surfaces that are hard to access (e.g., skyscrapers or bridges), and for time-constrained situations (e.g., post-disaster situations). TLS surveys typically comprise multiple scans from multiple positions; however, ALS surveys capture point clouds at different heights and distances without this manual operation. ALS is like TLS in the sense that the device/instrument also emits laser signals and calculates the distance, based on the time delay of the returned signals. 

The difference is that the mobile LiDAR device can travel up to 100 km/h and is free to rotate, which requires the need for a GPS receiver and an inertial measurement unit (IMU). The GPS allows the airborne device/instrument to record the exact location of the system to estimate where the device/instrument reflections are located on the scanned surfaces. The distance recorded by each returned laser pulse is subtracted from the recorded altitude of the system to yield the elevation of the scanned surface. To account for the tilt of the system, when the distance of the returned pulse is calculated, the IMU records the roll, yaw, and pitch of the system at each location. Most pulses are emitted at different angles, so the system also accounts for the pulse angle when calculating the elevation of each point. This information on the speed, angle, and rotation of the mobile system are all important for accurate elevation calculations and, therefore, for the acquirement of an accurate point cloud. 

Table 1 shows a detailed comparison of TLS and ALS in light of their applications in SHM [13,14,15,16,17,18,19]. 

## 3. Literature Review of LiDAR-Based SHM

**Figure 3:**
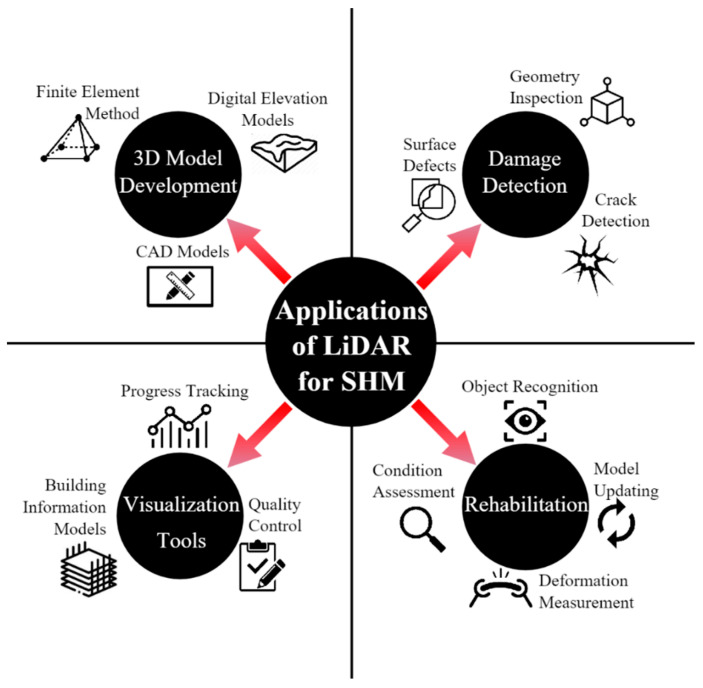
Applications of LiDAR for SHM.

Figure 3 summarizes various applications of ALS and TLS in a broad range of SHM applications in aging civil infrastructure.

### 3.1. Applications in Large-Scale Civil Infrastructure

#### 3.1.1. Road/Pavement

Mobility issues also limit the uses by which laser-based scanning can be conducted for pavement damage detection. The sensor must be directly attached to a vehicle to make the data acquisition process time-efficient. Ref. [8] reviewed the current methods and technologies for detecting damages (i.e., cracking, patching, potholes, surface deformation, and surface defects) on pavements. It was concluded that distresses that manifest primarily in height and depth differences require 3-D sensors to detect information in three dimensions. Ref. [22] developed a computational process to semi-automatically extract the irregularities of a concrete taxiway, based on DEM and TLS data. Once the DEM was constructed using an interpolation algorithm, the elevation profiles and the values of the longitudinal grade and transverse slopes were extracted. In another study, ref. [23] concrete slabs were evaluated, by calculating the faulting values between them using a TLS. With reference to the general plane, the altitude and the distance between the points of each slab-plane were computed to identify the critical sections. For each of these sections, the faulting values were determined by computing the distance along the normal to the general plane. 

Ref. [24] identified the potential causes of longitudinal cracking in Jointed Plain Concrete Pavement using TLS. Curling and warping were measured by the TLS at various sites, and the average curvature degree of the slabs was further computed. Ref. [25] proposed a data processing framework for identifying distress (e.g., potholes, swells, and shoves) in the pavement from MLS data. After the points belonging to the road surface were extracted, the height deviation of each point was computed for a modeled road surface. Two binary images were created from the DEM to analyze the positive and negative displacements. Finally, each segmented region of the full-scale pavement was classified, based on its severity levels. Ref. [26] used TLS data to scan a large concrete area to evaluate defects in the rigid pavement slabs. The acquired point cloud was cut into nine sub-clouds to represent each rigid slab, and the defects were characterized, based on three parameters (i.e., defected area, crack width, and intensity). 

#### 3.1.2. Bridges

LiDAR-based SHM assessment of bridges has predominately involved characterizing the geometric properties of various components (girders, deck, etc.). This information is often used to assess the vertical clearance underneath bridges to ensure that large trucks can safely pass beneath the structure. Various studies using LiDAR data have been conducted to ascertain the correlation between environmental properties, such as loading, temperature, and precipitation, on bridge deformation. A few studies have implemented LiDAR for the quantification of various structural damages, including spalling and crack detection. For example, ref. [27] proposed an automated method to recognize the mass loss of concrete bridges using TLS. The damage recognition was performed in three steps: (1) the point clouds were subdivided into sub-areas with a defined size, (2) a preliminary Gaussian filtering and parabolic fitting was performed for each point in each sub-area, and (3) each sub-area was classified as damaged or undamaged based on the corresponding curvature distribution. 

A LiDAR-based bridge evaluation for material mass loss quantification was explored by [28]. In a bridge-monitoring study, a phase-based laser system was used to compare both the distance and gradient-based damage quantification methods to detect the defected area. The results showed that combining the two approaches improves the identification and quantification capability of the LiDAR. In another study, ref. [29] developed an automatic and high-precision bridge clearance measurement technique based on TLS. The relevant part of the bridge was selected from the planar scan image to calculate the clearance, showing millimeter-level accuracy. In a similar direction, ref. [30] evaluated the impact of various parameters (i.e., elastic shortening, creep, shrinkage, relaxation, and thermal expansion) on bridge clearance measurements in a full-scale structure using periodic TLS scans. The applications, reliability, and evaluation methodologies of LiDAR technology for bridge health monitoring were assessed by [31]. Three sensitivity analyses (i.e., linear dimensional analysis, surface area analysis, and volumetric analysis) were performed on a full-scale bridge to test the system adjusting parameters, the range measurement ability, and accuracy of the scanner, as well as the automatic bridge inspection algorithms.

A novel procedure was presented [32] to measure the minimum vertical under clearance of bridges and to obtain the profile of prestressed concrete beams using photogrammetry and TLS surveys. To estimate the vertical under clearance and prestressed concrete beam cambers, a 3D curve-fitting algorithm was developed. After implementation on a full-scale bridge, high statistical correlation coefficients were achieved. Three case studies presented by [14] were used to validate the use of TLS scans for identifying various damage characteristics, such as loss of concrete, reinforcement corrosion, and surface erosion, on full-scale bridges. Under various truck loadings, the geometrical information (i.e., bridge elevation, span length, girder spacing, bottom flange width, and web height) was recorded by a LiDAR in [33]. The girder deflections were calculated by comparing the girder elevation coordinates of the scans with and without the weight of the trucks. In comparison to contact methods, the proposed approach proved to be superior in accuracy and accessibility, even in estimating the bridge’s natural frequencies. A fully automated TLS point cloud segmentation procedure was developed by [34] for the SHM of masonry arch bridges. A voxelization process first filtered out the redundant data from the point cloud, from which segmentation was performed by combining a heuristic method with image processing. To identify the individual structural elements, topological constraints were used to establish the spatial relation and order of the elements. Five data sets were used to test the proposed algorithm, showing coherent results with minor problems due to poorer point cloud quality.

Terrestrial photogrammetry was used by [35] to detect cracks in masonry arch bridges, while ground-penetrating radar was used to detect the hidden bridge elements. In combining this data, continuum damage and discrete models were constructed to determine the behavior of each arch under various loading situations. Two loading tests conducted on bridges by [36] were used to determine their vertical deformations using TLS. Rather than through individual points, the deformation analysis was conducted by one modeled surface serving as the reference surface. After the surface meshes were created, the zero-load epoch mesh was subtracted from each load epoch mesh. A new method called MCrack-TLS was established by [37], which combined image-processing procedures and TLS to assess 3D crack characteristics (i.e., width, length, orientation, and location) in concrete bridges. The TLS was used to identify and record reference target coordinates, removing the need for real coordinates of at least four reference targets required for traditional methods. A homography matrix, along with the acquired reference targets, was used to compute the image orthorectification. Testing on a concrete viaduct showed that the proposed method increased productivity and the quality of data compared to traditional methods.

An automated processing method of laser scanning data for the SHM of piers in masonry arch bridges was proposed by [38]. A full-scale bridge was first segmented, based on its structural elements (cutwaters, piers, spandrel walls, roadway, and vaults), so that the data size was reduced and each pier’s face could be analyzed individually. Structural faults were identified from the geometric parameters of the pier faces and their respective topological relation to the other bridge elements. SHM technology for a masonry bridge, using TLS data and Ground-Penetrating Radar (GPR), was presented by [39]. Comparing the TLS measurements with the historical drawings and plans of the bridge, the anomalies identified in the hyperbolic reflections provided information on the configuration of the bridge fillings. A structural deformation analysis framework was presented by [40] using TLS for applications on composite footbridges. Two mesh models were generated using the Fast Marching algorithm to check if a change occurred after a load was applied to the bridge. A crack identification and quantification approach for concrete structures were investigated by [41] using unmanned aerial vehicles. To establish an inspection map, a point cloud-based background model of the structure was first generated through a preliminary flight. From high-resolution images, regions with convolutional neutral networks for deep learning were applied for crack detection. A combination of 384 crack images and a model pre-trained through transfer learning were used for classification and localization for a full-scale bridge.

A deflection and deformation measurement application for bridges was proposed by [42]. The shape information model was first constructed using the improved octree data structure and the TLS data. This process reduced the size of the scan data for efficient memory usage and then estimated the deflection, based on the octree space division, which was then validated using LVDT. A new bridge inspection technique using UAV (Unmanned Aerial Vehicle) imagery point clouds was developed by [43]. To reduce errors in data (i.e., incomplete data, nonuniform distribution, outlier, surface deviation, and geometric accuracy), a triangular mesh and density map were constructed. The iterative closest point algorithm was applied with TLS data, and thickness, point distribution, and point-to-point distances were measured for a full-scale bridge. A reflector-based framework by [44] was applied for measuring long-term bridge displacement using LiDAR. The framework consisted of a reflector positioning strategy, measuring the reflector coordinates, and calculating the displacement. Having the reflectors as reference points allowed higher measurement accuracy, reduced scanning time, and there was no requirement to keep the same positioning of the LiDAR at different epochs. A prestressed concrete bridge was used to validate the accuracy of the proposed method. A TLS was used to construct a Digital Surface Model and identify the potential damage area in [45]. Ground-based microwave interferometry was applied to confirm where the bridge had damage, and an interferometry synthetic aperture radar technique was applied to analyze the causes of the damage of a full-scale bridge. 

A three-dimensional path planning method for LiDAR-equipped UAVs was introduced by [3] for inspecting bridges. The proposed method consists of three steps, (1) assigning low, medium, and high Important Values, based on moment and shear force values obtained from structural analysis, (2) selecting View Points of Interest for perpendicular and overlapping views, and (3) calculating the optimal collision-free path using Genetic Algorithm and A* algorithm. After being implemented on a full-scale bridge, it was concluded that the proposed path planning method decreased flight time, processing time, and workload while increasing visibility, reliability, and accuracy. A deformation monitoring process, based on TLS and ground-based radar interferometry data, was established and the process tested on a full-scale bridge by [46]. Using the TLS data from three epochs, the bridge’s vertical displacements were determined using a geometry-based approach, while another deformation was computed-based on deviation from the reference points. Comparison between the ground-based radar interferometry and the TLS showed close compliance with the results from both methods. In another recent study, ref. [47] proposed a method to obtain a displacement estimation of bridge structures using four laser scanning-based techniques. The vertical displacement was estimated by relocating the point cloud data in a 3D space and then dividing it in detail to search for the change in position of the leaf nodes. This study rearranged the points in a three-dimensional space, and nodes were created to calculate the displacement. Comparing the Grid, Tri, and LSP approaches of using distance estimation between points, the proposed method showed a decrease in the time required for displacement estimation, but an increase in the data processing time. 

To date, the use of TLS for quality inspection has been focused on identifying surface defects and the presence of water in structural members through utilizing differential geometry, RGB values, image segmenting, and gradient-based methods, as well as combining different image-based technologies, as summarized by [15]. For assessing structural performance, the common methods have been to construct geometric models as the basis for measuring deformations. The use of TLS and photogrammetric techniques for measuring the point-wise aspects (distances and lengths) was implemented by [20] for a historic suspension bridge. It was concluded that the distance from the bridge, as well as the complexity of the bridge, both highly influenced the accuracy of the measurements. Moreover, the hybrid surveying method acquired millimeter-level accuracy measurements, but the TLS performed better than the photogrammetric device. The B-spline surface method for the approximation, deformation analysis, and noise filtering of point clouds using TLS was quantified by [48]. After testing on a bridge under load, it was concluded that the mathematical approximation of the noisy point cloud was necessary for accurate computation, and the correlated noise impacted the distance computation for both the raw and approximated observations. A plane fitting approach was used in [5] to classify the points into six sub-plane categories (e.g., the bottom of bottom flange, edge of bottom flange, front edge effect plane, side of the web, bottom of top flange, and back edge effect plane) and fitted the points to the corresponding dataset using a linear least square method. Finally, Table 2 summarizes the devices used, type of assessment, and post-processing method for the LiDAR-based structural assessment of bridges.

#### 3.1.3. Tunnels

For underground tunnels, excessive-profile deformations are a significant concern as they often result in the collapse of the structure. Therefore, the majority of LiDAR or TLS-based inspections of these tunnels focus on ongoing data acquisition, which can be used to measure the change in profile deflection with respect to time. An approach for monitoring tunnel profile deformations with the use of multi-epoch LiDAR was established by [49]. The method was based on establishing point correspondences between the point clouds from different epochs and applying a minimum-distance projection algorithm to identify deformations. An autonomous technique for extracting tunnel cross-sections and removing non-lining points, based on TLS point clouds, was proposed by [50]. The first step was to estimate the tunnel boundary points using an angle threshold and 2D projection onto the X-Y plane. The direction of the cross-sectional plane was adjusted twice with the total least-squares method and Rodrigues’ rotation formula. Finally, an angle-based filtering algorithm removed non-lining points, based on morphological erosion. Validation on a real railway tunnel indicated that the proposed method was superior in accuracy compared to other methods, due to the consideration of the tunnel grade and the application of the filtering algorithm. A method to calculate the clearance of tunnels, based on mobile laser scanning, was developed by [51]. For the pre-processing step, the point cloud was segmented in the direction of the rail line, and the straight rail sections were identified. Based on the tunnel cross-section baseline and the individual cross-sections, the clearance inspection was carried out by calculating the distance between the cross-section and the testing rack.

A method for the automatic extraction of tunnel cross-sections was investigated by [52] based on mobile laser scanning to monitor deformations of a full-scale tunnel. First, the 3D point clouds were converted to a 2D surface, where the buffer of each cross-section was calculated by the K-Nearest neighbor algorithm. The iterative ellipse fitting was applied by combining the fitting of the sectional curve line with the denoising of the sectional point set. To reduce errors, the initial cross-sectional planes were rotated around their respective intercept points, and the optimal cross-sections were extracted. In [53], a processing method based on TLS for the change detection of the cross-sectional area of an underground gate road was explored. Three reference points were first established to reduce errors when data were compared from different scanner positions. A visualization software package was developed to visualize and analyze the deformations from different epochs. In another study, ref. [54] investigated the feature extraction of tunnel structures using TLS technology. The method comprised of segmenting the tunnel’s point cloud data into thin sections to acquire the projection plane for each section profile. Millimeter-level accuracy was achieved when the deflection of a subway tunnel was estimated. 

A new method was established by [55] that considered and corrected the effect of surface roughness on TLS intensity data for water leakage detection in underground tunnels. After the mean intensity values of each homogeneous region were computed, the distance and incident angle effects were corrected to improve the accuracy of the intensity data. The intensity image of the studied tunnel was generated, and the water leakage regions were detected based on an intensity threshold. Crack identification in tunnels by using TLS data was further explored in a study by [56]. After the point cloud was projected onto an image, an index method was used to indicate the position of each crack. To extract each crack, the standard deviation of the Gaussian template was used as a parameter, and a signal-to-noise ratio further extracted the smaller cracks. A crack detection method for tunnels was presented by [57], combining dilation and the Canny algorithm based on TLS point cloud data. The grayscale dilation process was employed to eliminate distinct textures in each image by using a disk-shaped structuring element. The Canny detector was adopted for crack edge detection, where the vertical crack widths were measured, based on the space of the two sharp peaks. The proposed method did not require determining Canny detector parameters, thereby being free of any major user intervention. Table 3 summarizes the devices used, type of assessment, and post-processing method for the LiDAR-based structural assessment of bridges.

### 3.2. Applications in Civil Structural Sytems

#### 3.2.1. Arched Structures

Similar to underground tunnels, excessive-profile deformations in arched structures are a significant concern for the continued integrity of the structure and must be monitored through TLS devices. An experiment was carried out by [58] on a brick and concrete arch structure under monotonic loads, based on TLS measurements. A surface model was created, and the deformation of the arch was calculated by subtraction of two epoch surfaces. The result was a sufficiently accurate dense point-wise representation of the deformation. A new TLS-based method, as proposed by [59], extracted the displacement size and direction of arched structures. A network and remapped point cloud were used to take the structural feature points and compare the exact points between two epochs. Analysis conducted by [60] quantified the deformation tendency of a composite masonry structure, based on TLS data extraction by the window selection method. The window-neighbor method first extracted the edge data. Moreover, polynomial fitting was applied to analyze and compare the deformation between each epoch during a loading test of an arch structure. Approximating TLS point cloud data for deformation analysis was conducted by [61] for arch structures through polynomial and B-splines surface models. To select an optimal parametric model, different adjusted surface models were compared through Cox’s and Vuong’s tests. It was concluded that the B-spline model was superior and performed better with more parameters.

A novel algorithm developed by [62] was implemented for the extractions of steel arches from a LiDAR point cloud of varying tunnel cross-sections (e.g., round and square). To extract the rock portion of the point cloud, slices were made along the X-axis, and the Differential Analysis for the Section Sequences of the Tunnel point cloud was applied to create curvature and height thresholds. Finally, the normal local saliency of each point served as the basis for extracting the steel arches from the rock surface. Results from testing on a full-scale tunnel showed the proposed method’s ability to extract the steel arches at 92.1% precision without manual assistance. TLS and the finite element method (FEM) were combined in [63] to construct an intelligent FEM model that could predict and simulate the deformation of arched structures. An experiment was conducted where a concrete arch was measured and inspected during different loading scenarios, and the resulting deformations and patterns were recognized to create an optimized FEM model. Table 4 summarizes the devices used, type of assessment, and post-processing method for the LiDAR-based structural assessment of arched structures.

#### 3.2.2. Historical/Heritage Structures

Preservation and rehabilitation of historic structures have become a key component of SHM, as many structures globally have reached, and exceeded, their service lives. Historic structures are often characterized by extreme fragility, and as such often require techniques that reduce physical interaction with the structure. Therefore, the use of non-destructive testing methods, such as laser-based scanning, allows inspectors to assess the structure without direct physical contact with the structure. A comparison of three methods (i.e., isodata algorithm, k-means algorithm, and fuzzy k-means algorithm) for characterizing historical building damage, using the intensity data from three different TLSs, found that the fuzzy k-means algorithm resulted in the best accuracy [64]. Evaluating the geometric characteristics (i.e., the global and local leaning and tapering angle, radius, local deviation from the circular shape, and the local curvatures) and the damage characteristics (i.e., masonry bulges, brick displacements, material loss, and cracks) of a historic masonry tower by [65] was conducted using TLS data. A toolbox was developed for the analysis of the point cloud, which provided an accurate georeferenced model of the structure. A case study on the SHM of a historical masonry building produced by [66] combined TLS and infrared (IR) thermal images for vulnerability analysis. TLS data were used for the 3D reconstruction of the structure, while the anomalies were detected from IR thermography. Metric features were assigned to the thermographic images, which mapped the anomalies and made them measurable. Finally, the vulnerability of the structural elements was assessed, based on the thermal defect being associated with geometric irregularity.

The vertical deformation of a minaret of a historical mosque was studied by [67] using TLS. Thirty-one horizontal sections of the minaret point cloud were created, which determined the deformation for each element from the calculated inclinations. Detailed deformation analysis of an ancient wooden structure was conducted by [68], combining three TLSs with different distances (long-range, middle-range, and handheld). After adding the coordinates of the target points into the coordinate system, the points from the three different scan locations were registered and geo-referenced into one dataset to perform the deformation analysis. Moisture content was quantified in [69] for heritage buildings based on autonomous TLS data acquisition. Once the point clouds were acquired, they were processed to the optimal level of reflectivity through an algorithm that determined if the incidence angle was within a certain threshold. The range of reflectivity was divided into a set of linear segments to create a model showing the moisture and humidity content, based on the change in the reflectivity index. Using TLS data, in a study by [70], a watertight mesh model was created and imported into the FE software to perform structural analyses. The deviation analyses used the point-cloud-based mesh model, to identify local deformations. The combination of the FE model and DA provided accurate and non-destructive gathering of information for structural analysis and monitoring. A numerical modeling strategy proposed by [71] implemented TLS point clouds for building FE models and SHM of historical buildings. The structural breakdown allowed a semi-automatic generation of the structural domain from the TLS. The validation of the strategy was performed on a historic fortress, which demonstrated increased level of automation and decreased computational time, compared to CAD-based modeling procedures.

Mobile LiDAR Systems were evaluated by [72] for the analysis of cultural heritage sites, based on a two-fold approach. A clustering phase consisted of computing the local curvature, defining the number of clusters, according to similar curvature values, and conducting a component analysis to reduce errors. Lastly, a weighted sampling was applied to each point inside a cluster, based on the extreme curvature values of the cluster and the associated feature (e.g., cylinder, plane, etc.). Testing on a medieval wall and an accuracy comparison using TLS showed reduced data acquisition time, but also reduced spatial resolution of the mobile LiDAR point cloud. A method to detect and localize deformation, specific to historic and heritage buildings, was proposed by [73], which used a generalized Procrustes analysis (GPA) and TLS data. The acquired data set was divided into subsets, and the GPA was applied by forming a matrix containing three-dimensional coordinates of each point of the point cloud. The deformation vectors and probability were computed, based on six transformation parameters. Various testing methods in historic sites showed the ability of the proposed method to reduce noise and improve the reliability and accuracy of the results. In another study, ref. [74] investigated the use of TLS for the post-fire inspection of a historic building. TLS scans were taken of the building before and immediately after the fire, and cloud-to-cloud registration techniques were used to identify the changes in the common features. The progressive decay and erosion of earthen heritage sites were assessed by [75] by combining multi-temporal TLS data and GIS. Using the Multiscale approach to the Model Cloud Comparison method, the surface change among different instances of the same feature was computed. Full-scale heritage walls were evaluated, and the deterioration values were imported into the GIS to express the occurrence of the variation.

The use of multi-temporal TLS data comparisons was explored in [76], based on the Multiscale Model to Model Cloud Comparison (M3C2) technique to detect material loss in ancient walls and buildings. Each instance of a compared feature was aligned to its reference point cloud, and the point normals were computed to detect change. The M3C2 method compared a sub-set of points based on a cylindrical projection from user-defined maximum depth and radius. Analysis of various damage assessment methods by [77] for heritage building elements was conducted, based on TLS. For the inner and outer walls, three different methods were tested: (1) approximating the point cloud centered on a point, (2) considering the plane as vertical and moving the origin point of that plane, and (3) considering a set of points within a certain distance to calculate the vertical plane. An automated deep learning model was developed by [78] for the surface damage detection of heritage sites using 2D images and 3D point clouds. After the data acquisition, Semantic Segmentation was applied to the images to remove the sources of noise and to generate a per-pixel classification of each image. The proposed method was validated after being tested on an unseen heritage site, proving accurate damage detection of complex heritage structures. Through a voxelating process, the point-to-point spacing was made uniform, such that the damaged areas of the point cloud showed a different point distribution [79]. Based on the eigenvalues, neighboring points covariance matrix, normal vector variation, and mean curvature to its closest neighboring points of each point, the damage was detected and re-evaluated. From the damaged areas of the point cloud, a density-based clustering algorithm was applied, which identified clustering structures for the categorization of the damage. Table 5 summarizes the devices used, type of assessment, and post-processing method for the laser-based inspection of historic structures.

#### 3.2.3. Concrete Structures

Laser-based scanning of concrete structures provides engineers in the SHM community with information about the severity of the damage. Furthermore, the 3D point clouds generated by these techniques provide quantifiable data about the level of cracking and spalling, which is easily obtainable through other optic-based devices, such as cameras or traditional surveying equipment. Three algorithms proposed and tested by [80] (i.e., range filtering, deviation filtering, and sliding window) were implemented for processing laser-scanned data for the assessment of surface flatness deviations. The range filtering and deviation filtering algorithms relied on range images with reversed orders of smoothing and deviation calculations, while the sliding window algorithm operated on 3D points to determine the deviation at each surface location. Testing on flat boards with defects of varying diameters and thicknesses showed that the sliding window algorithm offered the best detection performance. Two methods for quantitatively evaluating the damage in concrete structures, based on long-distance TLS, were proposed by [81]. Based on the region growing algorithm, the first method estimated the original shape of a structure before scaling and evaluated the total scaling depth. The second method was proposed using the iterative closest point algorithm, combined with a novel feature sampling technique, to evaluate secular changes in the scaling depth. An autonomous method by [82] was implemented for detecting and mapping cracks in concrete obtained from laser scanning surveying. To remove the noise elements, different filtering and image processing procedures were first executed. The probabilistic relaxation technique was applied to extract the crack track from the filtered image, and the pixel coordinates were transformed into global 3D coordinates. For mapping the crack, the results from the experiment showed an accuracy of 10–38 mm, compared to the total station survey. 

Comparisons were made by [83] between surface-based TLS measurements and the FEM model simulation of the displacement and loading of concrete cylinders. An error of less than 5% proved the feasibility of using TLS for the evaluation of FEM models. A novel approach developed by [84] integrated TLS with Building Information Modelling (BIM) to assess the flatness of concrete surfaces. The data processing was significantly automated by using the Scan-vs-BIM method, which matched the point cloud to the BIM model components. The Straightedge and F-Numbers methods were applied to control the compliance of the surfaces. The proposed method was successfully applied, as per the current standards for flatness specification and control in concrete slabs. An unexplored and novel technique to simultaneously localize and quantify spalling defects on concrete surfaces using a terrestrial laser scanner was conceived by [85]. Two defect-sensitive features (i.e., angle and distance deviation) were combined to identify defects, while a defect classifier was developed to diagnose the severity, location, and size of defects. A suite of scan parameters (e.g., scan distance, angular resolution, incidental angle) was investigated in the parametric study and numerical stimulations. The results showed that the proposed technique improves the autonomy, simultaneousness, and accuracy of detecting concrete defects.

A two-stage research project on reinforced concrete structures was proposed by [86] using laser scanning technology for crack identification and monitoring. Although the TLS data provided information on the swelling and surface height change of the concrete blocks, it was not possible to measure the cracks, due to the low scanning resolution and small crack widths. A surface normal-based damage detection method, as developed by [87], was implemented to detect and quantify damage types, such as cracks, spalling, corrosion, delamination, and rupture, using camera-integrated TLS. The defective areas were located using the model properties and grouped into individual damage clusters using a silhouette-based method. The quantitative information of the damage clusters was recorded using the presented damage area and volume computation strategies. The results from testing on a full-scale concrete test frame and a bridge showed that the proposed method automatically quantified and documented information, thus eliminating the need for human and computer interaction. TLS data was explored by [88] to identify and quantify areas of concrete loss in structures. The proposed approach was validated using accelerated laboratory testing to determine the feasibility of TLS for identifying crack initiation and subsequent crack growth, followed by crack monitoring in a forty-year-old reinforced concrete seawall.

A novel concrete crack detection method proposed by [89] applied an adaptive wavelet neutral network for the analysis of TLS data. A low-resolution fit of the entire 3D point cloud data was created, and further high-resolution analysis was performed only on regions with damage. Such an approach resulted in a compact representation of the TLS data, thus reducing memory usage and computational time. The detection of cracks on concrete structures by using image processing algorithms from the octree structure of TLS data was explored by [90]. Testing on a concrete dam showed that the proposed technique minimized the false recognition of cracks against stains, sediment, and structural joints. A concrete surface crack detection method by [91] combined the use of 2D images and 3D point clouds. The depth information from the laser scanners and gray information were merged at the pixel level. The improved Otsu algorithm yielded rough crack detection; therefore, denoising and connection of the cracks were required to refine the results. After several types of cracked concrete specimens were tested, the approach showed significantly better results than the single image or standalone point cloud methods. Integrating UAV-equipped LiDAR data and RGB images, ref. [92] identified and quantified cracks in concrete. The points within the point cloud were first associated with their corresponding structural element using the heuristic-based method. Once the crack patches were identified through a CNN-based classifier, an adaptive thresholding procedure of the grayscale intensity values aided in extracting the pixels with crack boundaries. Table 6 summarizes the devices used, type of assessment, and post-processing method for the laser-based inspection of concrete structures.

#### 3.2.4. Retaining Walls

Very few studies have been conducted regarding the inspection of retaining walls for damage defects using a laser-based scanning device. An automated framework was introduced by [93] for the feature extraction and displacement measurement of highway retaining walls using TLS data. The method was based on extracting the horizontal joints from the wall’s point cloud and using this data as a benchmark for detecting future displacements. Testing on a real-life dataset and 3D simulated models showed millimeter-level accuracy of the displacement measurements. A time-efficient method was developed by [94], intended for the morphological characterization of masonry blocks of a medieval wall, using TLS and MLS data. The 3D point cloud was reduced to 2D intensity images by computing the plane of projection and converting the 2D point cloud into a raster image. The watershed segmentation process involved differentiating the masonry blocks from the joints on the basis that the gray tone of each pixel represented the height of the surface. A change detection method, based on comparing baselines (a 3D line segment connecting two feature points in one scan) from the laser scanner data of two epochs, was developed by [95]. The target points (i.e., spherical targets, planar targets, or virtual points) were first extracted utilizing k-means clustering of the TLS intensity data. Comparing the two baselines from the different scans connecting the same features identified the changes in the x, y, and z directions. The proposed framework was validated during experiments on masonry walls, which showed reduced errors, without the need for a registration step.

Continuous Wavelet Transform (CWT) was applied to the 2.5D map using an estimate of the mortar joint width in [96]. The resulting scalogram showed the CWT responses for each pixel in the depth map. Furthermore, the dilation process delivered properly defined 2D stone segments to be re-mapped onto the 3D point cloud. The proposed method was tested on two structures for the evaluation of the changes in the individual stones, showing no sensitivity to global levels of flatness, waviness, curvature, and plumpness of walls. Rough surfaces were shown to have lower intensity values in [16], due to the laser signal being reflected several times. Also, the color of a surface affected reflectivity, with darker surfaces having an increased absorption of light and decreased energy of the return signal. A targetless and MLS-based SHM strategy for mechanically stabilized earth (MSE) walls was developed by [97]. The MSE wall façade was partitioned into individual planar faces, and the longitudinal and transversal lines along each face were established. The serviceability measures were determined based on the translational and rotational relationships, as well as the normal displacement between the corners of the fitted planes and each panel. The proposed method was validated when the MLS results from a segment of a full-scale MSE wall were compared with TLS and a profiler gauge.

A framework to reduce the size of point cloud datasets, while also detecting surface imperfections (i.e., cracks and cavities) and physicochemical issues (i.e., moisture, weathering, salt blooming, and biodeterioration), in building walls was explored by [98]. The point clouds were downsized using geometric and intensity data, both of which had thresholds associated with undamaged surfaces. From the remaining point cloud, the two types of data were analyzed, and defects were identified. A methodology for monitoring and detecting defects in levees was developed by [99], based on multi-temporal LiDAR point clouds and RGB images. The LiDAR data was used to produce two differential Digital Terrain Models, and the elevation changes were identified. The RGB images and calculated vegetation indices were used to search for changes in the levee land cover. A method for measuring the tilt and lateral displacement of retaining walls using mobile laser scanning was introduced by [100]. From the acquired point cloud, the retaining wall was extracted from the ground points through a binary classification. As opposed to computing point-to-point deviations, the anchored concrete panels were individually segmented and modeled with planar surfaces. Furthermore, changes in the plane’s key parameters indicated deformations between two epochs. Table 7 summarizes the devices used, type of assessment, and post-processing method for the LiDAR-based inspection of retaining walls.

#### 3.2.5. Roofs

Applications for laser-based inspection of roofs have been limited within the SHM field, in comparison to other analyses involving the entire 3D structure or various elements of the structures. As unmanned aerial vehicles with a sufficient payload capable of carrying a laser-based scanning device are required to conduct the survey, the relative accessibility of rooftop inspections becomes restricted. A segmentation method using laser data for damaged roofs was proposed by [101]. The individual points were grouped into planar regions, based on the assumption that undamaged roofs appeared as planar segments and collapsed roofs comprised of many small segments. The roofs were identified as intact or damaged based on the extracted features and a classifier trained from manually labeled segments. The automatic extraction of roofs through a data-driven approach by [102] integrated LiDAR data and multispectral ortho-imagery for improved city modeling and building inspection. The LiDAR data was divided into ground and non-ground points, where the non-ground points were further segmented to extract the roof planes. Using the ground mask, color, and textural information, the structural image lines were put into various classes (i.e., ground, tree, roof edge, and roof ridge). Various algorithms were applied to obtain a roof plane and remove planes constructed on trees. The proposed method was tested on two data sets and successfully extracted small planes and removed vegetation. An automated roof covering damage assessment method by [103], based on ground LiDAR, collected data in the aftermath of extreme winds. Experiments were conducted in a controlled laboratory where the k-means clustering algorithm was tested with different combinations of clustering evaluation criteria.

The structural damage of roofs was detected in [104], based on their 3D features extracted from only the post-event airborne LiDAR data. During the data pre-processing, the Digital Surface Model (DSM) for each building was created using post-earthquake LiDAR data, 2D GIS vector data, and the digital elevation model. For each building, damaged roofs were detected based on the 3D shape descriptor derived from the contour clusters of the DSM. The proposed method was validated using post-earthquake data, proving the better performance of the 3D shape descriptor compared to using geometric features for damage detection. A robust methodology to evaluate tornado fragility models with roof damage was conducted by [105] using post-tornado LiDAR data. The extracted geometric information (i.e., height, slope, pressure zones, and distance to tornado path) for each roof was used to produce fragility curves for roof sheathing failures. Based on the LiDAR data, the tornado wind speeds and building damage were calculated and compared with the values estimated by the fragility curves. 

#### 3.2.6. Post-Disaster Reconnaissance

Natural disasters, such as earthquakes, landslides, hurricanes, and floods, often create logistical constraints for structural inspection, thus increasing the difficulty in performing human-based inspections. As such, non-contact devices that can be deployed at a distance from the damaged structure, such as LiDAR devices, are increasingly being implemented for post-disaster reconnaissance of structures and hazard regions. Two processing strategies for post-event ALS data were compared by [106] to automatically detect collapsed buildings. A segmentation algorithm first identified the planar regions, and the geometric and radiometric attributes were calculated. The rule-based classifications strategy identified collapsed buildings, based on each segment’s attributes and the corresponding thresholds. The Maxent classifier detected collapsed buildings, based on the maximum entropy modeling. Both strategies were relatively accurate, although the rule-based classifier required some manual input of the threshold values, and the Maxent classifier was highly dependent on the availability of precise training data. The advantages and limits of using remote sensing for post-earthquake damage extraction were summarized by [6]. They concluded that the open issues were the definition of a damage scale based on the data, the use of data fusion techniques, and the use of crowd mapping procedures. A TLS-based method to rapidly evaluate earthquake-induced damages to buildings was proposed by [107]. The point clouds were first interpolated to create the primitive plane. The computation of the differences between the primitives and the points produced morphological maps. If multi-temporal data was available, difference maps, as well as the detections of changes of the primitives, were obtained. The proposed procedure was validated by three case studies being conducted on earthquake-damaged structures, all with varying multi-temporal data available.

An evaluation of multi-temporal and mono-temporal methods for identifying earthquake-induced building damage from optical, LiDAR, and SAR data was reviewed by [7]. The common technique using pre- and post-event LiDAR data involves comparing multi-temporal 3D building models to assess the basis of the damage classification. With only post-event data available, algorithms have been developed for automatic plane detection from LiDAR data. A mobile LiDAR was employed by [108] for post-disaster data collection and to develop mapping approaches for damage assessment. From the data collection after a hurricane, the laser scans and imagery were tied together, employing post-processing of the trajectory of the vehicle. The change was detected by comparing the pre- and post-event data, and it was concluded that the proposed approach yielded more detailed information and analysis than traditional methods. The use of laser scanning technology for civil engineering laboratory tests and reconnaissance of earthquake-damaged structures was discussed by [109]. A method for the delineation of earthquake-damaged buildings from an image-based 3D point cloud (Blom-CGR) was developed by [110] and identified the broken elements of the buildings that led to the gaps in the point cloud. The gaps were then classified into four categories (i.e., occlusion, failure in 3D point generation, opening in architectural design, and damage) based on their surrounding damage patterns. 

A framework was introduced by [111] that estimated the damage of structural frame members using information from post-earthquake point clouds and the pre-damaged BIM model. The scanner’s point clouds were registered in the local cadastral coordinate system and segmented to construct planar surfaces. Objects from the as-built BIM model were compared to the segmented point cloud, and any deviations (i.e., cracks or breaks) were identified and updated in the as-damaged BIM model of RC frames. The Structure from motion (SFM)-based dense reconstruction method for conducting a post-hurricane residential building damage assessment was investigated by [112]. The generated 3D point clouds obtained from mobile LiDAR data were compared with SURE and Autodesk’s 123D Catch. A quantitative approach by [113] implemented airborne LiDAR data for the identification of post-earthquake building damage for buildings with different roof types. To extract the severity of building damage, the study utilized surface normal algorithms and the ratio of the standard deviation to the mean absolute deviation of the angle between the surface normal and the zenith. The authors concluded that the proposed method effectively estimated damage independent of the roof style and without the need for pre-earthquake data. Similarly, ref. [114] presented an automated method for the segmentation and damage assessment of post-earthquake buildings using airborne LiDAR obtained from 1953 buildings.

A damage evaluation framework by [115] integrated LiDAR scan data and photogrammetry technologies. The damaged estimation was enhanced by a joint analysis being conducted on the results from both the image-based method and the 3D coordinate method. A study using LiDAR scan data of an earthquake-damaged wall of a building proved that the proposed method significantly improved the accuracy of the damage assessment results. The use of LiDAR data to detect damaged buildings by using digital surface models (DSMs) before and after an earthquake was investigated by [116]. A total of 26,128 buildings were evaluated based on three parameters: the average change in height, its standard deviation, and the correlation coefficient between the two DSMs. Results showed that the most influential factor for damage detection was the change in average elevation. An automated approach for the post-disaster structural damage evaluation of major building envelope elements (i.e., wall, roof, balcony, column, and handrail) was developed by [117] using mobile LiDAR data. The building was first semantically parsed into segments, and damage detection was conducted to extract the semantic structural damage information. The study was the first automated building component-level damage assessment with high-resolution point cloud data sets.

A novel airborne LiDAR-based approach by [118] was implemented for the assessment of a wide scale of post-hurricane building damage. The building objects were first extracted from pre-event and post-event data, and a cluster-matching algorithm was used to compute the differences between the extracted building objects from the multiple-temporal data sets. On a hierarchical basis, the damage was estimated based on the damage indicators. Compared to other methods, the proposed approach effectively-recognized building objects extracted damage features, and characterized the extent of damage all at the individual building level. A comprehensive framework by [119] detected various damage types (i.e., multilayer collapse, outspread multilayer, pancake collapse, upper stories collapse, heaps of debris, collapse of all floors, inclined plane buildings, and inclined to overturn collapse) based on post-event LiDAR data. One conventional and two novel texture extraction strategies were used to generate the textural features. An improved Vosselman filtering method identified pancaked buildings, and the inclination angles were estimated from LiDAR data. From ambient vibration results analyzed in [120], modal parameters were extracted, and an FE model was subsequently created and updated. The LiDAR data was used to quantify the defects and to compare with the estimated damage from the vibration measurements, which showed good agreement with the developed model. Table 8 summarizes the devices used, type of assessment, and post-processing method for laser-based post-disaster reconnaissance.

#### 3.2.7. Other Structural Members

A recent study by [121] consisted of deriving beam-deflection equations using beam mechanics based on two TLSs. Two experiments on concrete and timber beams were analyzed using the least-squares solutions before and after the statistical testing. The modeling of raw point data for surface representation was undermined by the modeled TLS data yielding significantly higher accuracy than the TLS’s coordinate precision. A displacement measurement model was presented by [122] for SHM using TLS. After the shape information was acquired from the TLS, the base vectors were generated using the least-square method. The vectors were used to transform from the TLS coordinate system to the structural coordinate system. The displacements of a steel beam were computed by acquiring the deformed shape information, transforming the coordinates, and using the least square method. The proposed model improved measurement accuracy, while the maximum deflection was ~1.6% of that obtained from traditional LVDTs. 

The feasibility of TLS to perform structural change and deformation analysis was outlined in a study by [123], which proposed a method to apply TLS in a large-scale experimental setting. During the analysis of the point clouds, the specimen was sliced by the element, and the cross-sections were used to determine the volumetric change (i.e., bulging, compression, and spalling). In comparison to conventional means, the presented approach allowed for increased visualization of the specimen change and faster identification of damage. A study on two 2D reinforced concrete frames by [124] tested the use of TLS for measuring structural deformations under lateral loading. The acquired point clouds were first filtered and converted into 3D coordinates. Applying the root mean square errors of transformation to the coordinate differences identified the deformed points.

A review of recent methods for change detection was conducted by [125] using mobile and static laser scanning data. The main challenges that were identified were point cloud registration, varying measurement geometry, varying positions of data acquisition, and temporary objects present in scans. Furthermore, it was emphasized that the signal-to-noise ratio should be computed to evaluate the redundancy of the point clouds in the processing steps. A novel first-ever algorithm by [126] was designed to automatically measure the deformation from torsion and the deflection from the bending of metal beams by using TLS, as well as to determine the location of maximum stress. The process consisted of segmenting the beam flange and using polynomial surface fitting to eliminate noise and errors. The planar surface was fitted to the point cloud, where the deformations and stresses were calculated using the LiDAR data. The proposed methodology was tested on a steel beam under various loading situations, proving that it yielded accurate results. An algorithm proposed by [127] used TLS data that measured and modeled the deformation of metal beams using segmented point clouds and the polynomial fitting of the deformed curved surfaces. A three-stage process model for the deformation analysis of structures was developed by [13], based on a review of recent TLS-based methods. The change detection methods have, thus far, primarily involved computing and comparing the distances between point clouds or fitted surfaces of two epochs (i.e., point-to-point, point-to-surface, and surface-to-surface).

Alpha-Shapes were applied in [128] to determine the crack outlines in timber beams, and a minimum area was fixed to delete any false cracks. The geometric characteristics of each crack (i.e., main direction, area, crack centroid, length, and maximum width) were obtained for future comparisons and monitoring. Testing on laboratory specimens and a timber roof validated the suitability of the proposed algorithm for finding cracks and detecting growth between epochs. The deformation behavior of arch and beam structures under static loads, based on TLS and the surface analysis method, was explored by [129]. The scan data from each structural element was used to create a polynomial fit of the beam and a surface fit of the arch to be compared. The displacement was calculated from the deviations between the scans of different epochs. A 3D point cloud change analysis approach was presented by [130] for detecting changes in structural inspections. Once two 3D point clouds were spatially registered, comparisons were performed using the point-wise distance estimations to track and quantify displacements on a per-point basis. To overcome distorted measurements, a statistical sampling technique was used to extract a measurement from a localized set of per-point measurements. A series of flexural tests were performed to evaluate the proposed approach, showing that it is an accurate and automatic way to track small movements of structures over time. Methods were explored in [131] for providing quantitative estimations of textural damage (i.e., tile spall off, metal rusting, and water staining) using LiDAR data. The intensity and RGB model information were first used for the clustering analysis, and textural damages were detected by applying four data clustering algorithms (i.e., k-means, fuzzy c-means, subtractive clustering, and density-based spatial clustering). The results showed that the RGB information was not effective in detecting textural damage and that the k-means and fuzzy c-means algorithms gave better clustering performance and computational efficiency.

A novel 3D surface descriptor was created by [132] for the automatic identification and classification of surface defects using point cloud data from a 3D reconstruction system. The local models on each 3D surface were first estimated and then used to calculate the differences between the model’s normals in a local region. The primitives were projected onto a plane, where the defects were recognized from the geometrical features of the 2D images. Using a support vector machine, the defects were classified into three categories (i.e., holes, bumps, and cracks) with improved robustness to noise. Laser scanner data was used to generate a 3D Surface mesh in [133] for welding inspections, and the photogrammetric data generated a 3D point cloud both for quality assessment purposes. It was concluded that the accuracy of both technologies was similar; however, the macro-photogrammetric technique yielded superior geometric and radiometric resolution. A method proposed by [134] was applied to measure the strain and deformation of a steel plate using discretized 3D coordinate data of a LiDAR. The deformation shape of the plate was first modeled by a high-order polynomial function through regression analysis. Using a finite element method, a strain measurement model was generated, from which the strain on the steel plate was estimated.

A method for the inspection of piping components was created by [135], based on comparing the as-built TLS and the as-designed CAD data. The geometric parameters were recognized from the acquired point cloud via a normal-based region growing segmentation and the RANSAC method. Performing geometric parameter comparison and distance-based deviation analysis on each pipe segment identified differences from the as-designed model. The use of TLS for identifying and measuring surface imperfections and distortions of precast concrete elements was evaluated by [136]. The proposed techniques for evaluation were validated through precast concrete bridge deck panels. An approach for the Finite Element (FE) model updating damaged structures was developed by [137] and used to compare the results to LiDAR data. A two-story masonry building was measured at its reference state, where both tuned and un-tuned FE models were created to test the effects of the material properties and existing damage on updating FE models. After forced vibration tests were performed with four exterior walls being removed, the final FE models showed good accuracy, compared to the LiDAR results.

The use of a TLS, compared to Close-Range Photogrammetry (CRP) based on the Structure from Motion (SfM) algorithm, was evaluated by [138] for measuring structural deformations. A case study was conducted on two reinforced concrete beams subjected to four-point bending loading conditions. The point clouds at various loading stages were compared by using the Mesh to mesh and modeling with geometric primitives methods. The modeling approach yielded better results than the Mesh to mesh, while the CRP results were more accurate than the TLS, due to the short capturing distance and dimensional scale of the images. Using the RGB values of the TLS data, 2D images were created and segmented for precast concrete elements (PCEs) in [139]. Based on the edge image, the active window method was applied, which extracted the important data within each image cluster. The RBNN algorithm was used to avoid under-segmentation, and the segments were reconstructed to avoid over-segmentation. Compared to other methods, the proposed technique improved the data acquisition efficiency for the quality inspection of PCEs. An automated framework for the extraction of structural components (i.e., column, slab, and rebar) from point cloud data for progress monitoring and compliance control during construction was proposed by [140]. From the registered point clouds, the planar and linear features were extracted and semantically labelled into various categories. In comparing the as-built and the planned BIM, deviations were identified and visualized. Five sets of TLS point clouds from a construction site were used to test the proposed method, which showed success in component extraction and removal of redundant surfaces.

The performance of different non-contact sensors (e.g., 3D laser scanners, photogrammetry, and 2D cameras) and algorithms (e.g., feature extraction algorithms, Alpha-shape algorithm, and local entropy-based thresholding algorithm) was reviewed by [9] for the quality assessment (i.e., dimensional, surface, deflection, and deformation quality assessments) of buildings and civil structures. To enhance the accuracy and applicability of quality assessments, data fusion-based approaches (e.g., laser scanning data with vision data, 2D images with depth data, and image data with GPR sensors), as well as more robust and generic techniques, were suggested. Novel methodologies were developed by [141] for both the object and damage detection of common structural members from 3D point cloud data. New skeleton and graph-based object detection approaches were applied to the segmented point clusters, which involved taking the connectivity information to find the surfaces apart from one another of the same object. By comparing the fitted objects from test specimens and test-bed bridges with the acquired point clouds, defects were located and quantified. An algorithm presented by [142] used TLS for measuring structural deflection and damage. The TLS data was acquired for the loading and unloading scenarios, and the plane was fitted for the point cloud using a robust genetic algorithm. The scanner coordinates were transformed into structural coordinates to be curve fitted for the loading case. The deflection was estimated and compared between LVDTs. The strain and deformation of a steel plate under lateral pressure using LiDAR data were evaluated by [143]. Through a specific interpolation procedure, the point cloud was converted into a 3D mesh model, and the displacements from the initial shape were computed, followed by the strain calculations. Table 9 summarizes the devices used, type of assessment, and post-processing method for the laser-based inspection of structural elements.

## 4. Existing Challenges of LiDAR in SHM

Although LiDAR-based structural assessment has received increased attention in the SHM field over recent decades, there are still limitations related to the non-contact sensing process. The most challenging step in LiDAR-based monitoring is acquiring a point cloud that is high quality, dense, and uniform. Errors in the point cloud can yield redundant data or gaps in the point cloud, thus also affecting the application and accuracy of algorithms for detecting structural damage. The quality of data that the MLS or TLS device acquires is highly dependent on several parameters, including the following: (1) the presence of occlusions or redundant objects, (2) surface properties, (3) scanner positioning, (4) scanner specifications, and (5) the environmental conditions. 

The first major challenge to acquiring a point cloud that is ready for structural analysis is the large amount of redundant data that must be filtered. Dependent on the application, this may include vegetation, people, and non-structural components of the structure or surface being studied. The presence of this redundant data is problematic, due to the direct correlation between computational times with the larger datasets. Also, redundant data can act as obstacles in the scans that prevent the acquisition of data of the objects of interest. One of the main disadvantages of their scanner-based bridge evaluation was the presence of occlusions that prevented the entire scan of the target structure in [81]. Limited surficial scans were also conducted by [109], which employed laser scanners for post-earthquake damage evaluation, but only a few scans were taken of exposed and visible structural members. 

Surface properties also have a significant impact on the quality of the LiDAR scans. The surface smoothness and general features of the surface may enhance noise within the point cloud, making it difficult to differentiate between surface deviations that may or may not impact the structural integrity of the element. A method for identifying cracks in concrete bridges was developed by [37] but found that there was limited capability of the proposed method when there were stains that hid the cracks and interrupted the crack boundaries. Post-processing techniques, as indicated by [41], may be implemented to reduce the negative effects of surface-based contamination for LiDAR-based crack quantification. However, the evaluation of the structural health of heritage structures continues to prove challenging, as concluded by [77], as the larger amount of surface flaws acquired over time makes it even more difficult for damage and change detection using LiDAR to provide accurate results. 

The quality and completeness of point cloud data are also heavily correlated to the relative positioning of the scanning device. TLS point clouds can be particularly difficult to obtain due to overlapping or targetless scans if the field of view or incident angle of the sensor is not positioned strategically. The proposed method was not suitable for curvature-based analysis if the noise exceeded a certain threshold in [27]. Similarly, ref. [31] observed that a larger scan angle increased the error of selecting the same interest point on surfaces from different scan images, thus also increasing the error for target dimension measurement. Moreover, the distance from the intended targets also impacts the accuracy of damage detection methods implementing raw point cloud data. For damage or change detection, ALS is often subject to larger scanner distances and unfavorable scanning angles. Airborne sensors were used for post-disaster assessment but found that the scanner could only acquire information on the roofs and lateral walls of buildings from its position above in [6]. An unmanned aircraft system was implemented for the structural assessment of a bridge and used a planned flight path to ensure that the positioning and orientation uncertainties did not affect the data characteristics in [144]. 

Scanner specifications, such as the device’s range, the mobility of the device, and the general settings, are important limitations to be considered for LiDAR-based SHM. For surface damage detection, it is a common issue that the scanning precision of the LiDAR device does not allow the identification of smaller defects in the range of several millimeters [27,81]. When comparing the scanning specifications of TLS and ALS, there are limitations to using each type of scanner based on the scanner’s requirements. ALS requires the integration of a GPS, thus rendering GPS-denied areas an issue for acquiring point cloud data, as described by [145]. However, ref. [40] found that using TLS for change detection had some disadvantages as well, such as the sensitivity to scanner positioning, fluctuations in point cloud density, and complex data processing. Additionally, ref. [43] employed both TLS and MLS in their research and underlined the high noise levels, difficulty in key point matching for narrow features, and a longer 3D reconstruction process when using MLS. 

Finally, the environment in which the LiDAR scans are taken can create obstacles to performing structural analysis on a high-quality point cloud. The primary environmental factors include surface illumination (e.g., sunlight and shadows) and weather conditions (e.g., temperature, humidity, wind, rain, and visibility). Shadows projected by buildings or vegetation and sunlight contamination all contribute to increased noise within a point cloud. LiDAR devices work by bouncing laser beams off surrounding objects, therefore, rain, snow, fog, or dust do not permit scans with high resolution. ALS is often subject to poorer scanning angles, non-uniform point clouds, and high exposure to the environment; therefore, airborne LiDAR data can have reduced quality. While using mobile LiDAR, poor surface illumination or windy environments both highly affected the quality of the point cloud data and the ability of the proposed algorithms to detect cracks in [145]. 

## 5. Future Research Directions of LiDAR-Based SHM Technology

From the literature review, research being conducted using MLS, TLS, and other LiDAR-based devices over the past two decades has been extensive, resulting in many advancements to point cloud data capturing and assessment. However, there are still limitations for point cloud acquisition, data processing, and feature extraction that current methodologies have not yet addressed. During the data acquisition phase, noise from environmental factors and low point cloud resolution, due to obscured visual angles, are still prevalent within this research domain. As a result, data acquisition is often inaccurate or constrained by the physical parameters of the site under investigation. Future research should focus on developing optimization methods for parameter selection to automatically reduce noise and occlusion during data acquisition, depending on the type of structure. Additionally, best practices for choosing scanning locations and distances should be developed to ensure the completeness of the scanned data and improve the efficiency of the scanning process.

Though extracting 3D point cloud from structures is relatively accessible using LiDAR devices, the complexity and size of the extracted data make the post-collection processing a complicated and time-extensive endeavor. Most datasets can be reduced based on the localization of the analysis that is to be conducted; however, current processes require the manual manipulation of the point cloud to extract relevant information to construct a 3D model. Methods for the automatic conversion of raw point cloud data into a 3D model should be explored to maximize the efficiency of post-collection data processing. Furthermore, the robustness of these data processing techniques must be explored to ensure accurate 3D models are extracted from the existing point cloud data. Domain-specific knowledge extracted from objects scanned by LiDAR can be incorporated in the data post-processing stage to enhance the accuracy of the 3D point cloud conversion. Therefore, future methods should focus on robust methods that include geometric and physical parameters of structures for point cloud processing.

Following the processing of point cloud data, the 3D models are used to extract quantitative and descriptive data about the damaged structure through the use of statistical models and Artificial Intelligence (AI) techniques. The quality of information extracted from the model is directly correlated to (1) the quality of the data implemented and (2) the robustness of the analytical technique used for the investigation. Though many AI techniques and statistical models have been applied for the evaluation of point cloud-based models, domain adaptability remain a prevalent issue. Domain adaptation is the ability of an algorithm that is trained on a source domain (i.e., cracked concrete walls) to perform on a related target domain (cracked concrete columns). Therefore, future research should improve the domain adaptation of existing classification techniques to improve robustness for damage detection for complex structures with a variety of damage types. Furthermore, the accuracy of the quantification of physical parameters of damages, such as cracks, should be improved for cases where the damage features are small (<1 cm).

## 6. Conclusions

The increased prevalence of natural disasters in conjunction with global infrastructure reaching the end of its service life has spurred the SHM community to develop efficient monitoring and inspection techniques for structures. The diverse and unique challenges presented by various structural components, such as roads, bridges, retaining walls, buildings, and various other structural elements, have instigated an expanded research effort into sensor technologies for SHM. Non-contact sensing techniques, particularly LiDAR-based analysis, have received growing attention with the SHM community recently for their damage detection abilities through the generation of 3D point clouds. This paper provides a comprehensive review of LiDAR-based SHM techniques and the analysis of structural damages using laser-based point cloud data. The development of algorithms for bridges, tunnels and arch structures, post-disaster reconnaissance, historical and heritage structures, masonry surfaces, roofs, pavement and roads, structural elements, and walls have been summarized, and the existing limitations have been discussed. For LiDAR-based SHM, future research should focus on the development of more computationally efficient, autonomous models that are connected to the physical domain they are derived from.

## Figures and Tables

**Table 1:** Comparison of advantages and disadvantages of TLS and ALS.

	TLS	ALS
**Advantages**	More detailed and precise point cloud. Better control over the captured point cloud; less redundant data to filter out during the post-processing stages. TLS-based technology is less complex to operate than ALS, which incorporates GPS and IMU elements.	ALS is highly automated and requires less manual movement of the instrument. It requires less emphasis on each scan’s angle and target, as it can be maneuvered at different heights off the ground, which captures more points. More points are captured than in typical TLS scans, therefore, larger surface areas can be covered by the scans. It can capture harder-to-reach targets (e.g., roofs of buildings) and larger areas in a shorter period.
**Disadvantages**	TLS is highly manual and requires the movement of the scanner to capture the intended target(s). There is less flexibility in the device’s mobility; thus, incomplete or obstructed scans are common. Larger point clouds take longer amounts of time to acquire. TLS devices often are unable to reach remote or hard-to-reach locations.	Pre-planned flight paths are often required to reduce the amount of redundant point cloud data. These devices are highly dependent on weather conditions, due to consistent speeds being required for uniform point clouds. With an abundance of data captured, ALS point clouds require more complex and time-consuming post-processing methods.

**Table 2:** A comprehensive summary of laser-based assessment techniques for bridges.

References	Laser-Based Scanning Device	Type of Assessment	Post-Processing Method(s)
[3]	N/A	Full-scale inspection	Important value analysis with genetic and A* algorithms
[5]	FARO Focus 3D	Geometric assessment	The plane fitting least-squares method
[14]	N/A	Material mass loss, erosion, and corrosion	DGC and MMSET methods
[15]	N/A	Full-scale inspection	
[20]	Leica TC2002	Geometric assessment	Hybrid photogrammetric methods
[27]	Riegl LMS Z-420i	Material mass loss	Curvature distribution
[28]	FARO LS 880HE	Material mass loss	Distance and gradient-based
[29]	FARO LS 880HE	Bridge clearance	LiDAR bridge evaluation (LiBE) method
[30]	N/A	Impact of parameters on bridge clearance	Correlation analysis
[31]	N/A	Bridge health monitoring	Sensitivity analysis and LiBE method
[32]	Riegl LMS Z-390i	Bridge clearance	3D curve-fitting algorithm
[33]	FARO LS 880HE	Geometrical assessment under various loadings	The difference in elevation data
[34]	Riegl LMS Z-390i	Full-scale inspection	Voxelization and topological constraints
[35]	Leica TCR1102	Geometric reconstruction	Continuum damage and discrete models
[36]	Leica ScanStation C10	Structural deformation	The difference in surface profiles
[37]	Riegl LMS Z-390i	3D crack characterization	MCrack-TLS
[38]	Riegl LMS Z-390i	Pier analysis	Geometric and topological analysis
[39]	FARO Focus 3D X330	Full-scale inspection	Comparison of scanned data and drawings
[40]	Leica ScanStation C10	Structural deformation	Fast marching algorithm
[41]	N/A	Crack identification	Region-based Convolutional neural network
[42]	Leica ScanStation C5	Structural deformation	Octree space partitioning (OSP) algorithm
[43]	Leica ScanStation P20	Full-scale inspection	Closest point algorithm
[44]	Riegel VZ-1000	Structural deformation	Reflector coordinates analysis
[45]	Riegel VZ-1000	Damage detection	Digitial surface model analysis
[46]	Leica ScanStation2	Structural deformation	Geometry-based analysis
[47]	N/A	Structural deformation	Various displacement estimate methods and OSP
[48]	Zoller + Frohlich Imager 5006H	Structural deformation	Hausdorff distance and averaged derivation comparison

**Table 3:** A comprehensive summary of LiDAR-based SHM for tunnels.

Reference	Laser-Based Scanning Device	Type of Assessment	Post-Processing Method(s)
[49]	Zoller + Frohlich 5010 TLS	Profile deformations	Minimum-distance projection algorithm
[50]	FARO X130	Tunnel cross-section	Least squares, Rodrigues’ rotation and angle-based filtering
[51]	N/A	Tunnel clearance	Differences between Sequential Profiles
[52]	Regel VUX-IHA	Profile deformations	K-Nearest Neighbor and iterative ellipse fitting Algorithm
[53]	N/A	Tunnel cross-section	Differences between profiles
[54]	N/A	Profile deformations	Circular filtering and RANSAC algorithm
[55]	Riegl VZ-400i	Water leakage detection	Intensity thresholding method
[56]	N/A	Crack identification	Index and Gaussian template methods
[57]	N/A	Crack identification	Dilation and canny algorithm

**Table 4:** A comprehensive summary of LiDAR-based SHM for arched structures.

Reference	Laser-Based Scanning Device	Type of Assessment	Post-Processing Method(s)
[58]	Zoller + Frohlich Imager 5006 and Leice Laser	Profile deformations	Differences between sequential profiles
[59]	Zoller + Frohlick Imager 5006	Profile deformations	Differences between sequential profiles
[60]	Zoller + Frohlick Imager 5006	Profile deformations	Window-neighbor method and polynomial fitting
[61]	Leica AT960LR	Profile deformations	Polynomial and b-splines models
[62]	P + F R2000 UHD	Tunnel cross-section	Differential analysis and normal local saliency
[63]	N/A	Profile deformations	Finite element methods

**Table 5:** A comprehensive summary of laser-based assessment techniques for historic structures.

References	Laser-Based Scanning Device	Type of Assessment	Post-Processing Method(s)
[64]	FARO Photon, TRIMBLE GX200 and Rieg Z-390i	Damage analysis	Isodata algorithm, k-means algorithm, and fuzzy k-means algorithm
[65]	ILRIS 3D	Geometric and Damage Analysis	MATLAB Octave Toolbox
[66]	Zoller + Frohlick Imager 5010c	Damage analysis	Thermography and 3d model analysis
[67]	Trimble GX 200	Deformation analysis	Inclination calculations
[68]	Riegl VZ 1000, FARO Focus 3D and Handyscan 3D	Deformation analysis	Digital reconstruction
[69]	Leica HDS-3000	Moisture measurement	Reflectivity-based model
[70]	FARO Focus 3D S-120	Deformation analysis	Finite element model and deviation analysis
[71]	N/A	Damage analysis	Finite element model
[72]	LYNX Mobile Mapper	Damage analysis	Clustering and weighted sampling
[73]	Leica ScanStation P20, Leica T830, and Leica P40 ScanStation	Deformation analysis	Probability analysis of deformation vectors
[74]	Leica ScanStation 2, Leica ScanStation C10, FARO Focus 3D 120, and Zoller + Frohlick Imager 5010c	Damage analysis	Cloud-to-cloud Registration technique
[75]	N/A	Damage analysis	Multiscale model cloud comparison
[76]	FARO Focus S120	Damage analysis	Multiscale model to model cloud comparison
[77]	Leica RTC360	Damage analysis	Point cloud analysis
[78]	N/A	Damage analysis	Region-based Convolutionalneural network
[79]	FARO Focus 3D X120	Damage analysis	Density-based clustering algorithm

**Table 6:** A comprehensive summary of laser-based assessment techniques for concrete structures.

References	Laser-Based Scanning Device	Type of Assessment	Post-Processing Method(s)
[80]	N/A	Surface roughness	Filtering and sliding window
[81]	Riegl VZ-400	Scaling detection	Region growing algorithm
[82]	Leica C10 TLS	Crack detection	Probabilistic relaxation technique
[83]	N/A	Displacement measurement	Finite element model
[84]	FARO Focus 3D	Surface roughness	Scan-vs.-bim method
[85]	FARO Focus 3D	Spalling detection	Angle and distance deviation with classifier
[86]	Trimble CX	Crack detection	Point cloud analysis
[87]	FARO Focus 3D and Photon 80	Crack, Spalling, Corrosion, Delamination and Rupture Detection	Silhouette-based Method
[88]	Trimble TX5 3D	Spaling detection	Multiscale model to model cloud comparison
[89]	Trimble TX5 3D	Crack detection	Low and high-resolution fit
[90]	Leica ScanStation C5	Crack detection	K-means clustering, median filtering, and otsu’s binarization
[91]	Reigl VZ-2000 and HandySCAN 700	Crack detection	Otsu’s binarization
[92]	Velodyne VLP-16	Crack detection	Convolutional neural network

**Table 7:** A comprehensive summary of laser-based assessment techniques for retaining walls.

Reference	Laser-Based Scanning Device	Type of Assessment	Post-Processing Method(s)
[16]	N/A	Moisture detection	Reflectivity analysis
[93]	Riegl VZ-400	Displacement measurement	Difference analysis
[94]	Riegl LMS Z-390i	Morphologic characterization	Raster image and watershed segmentation
[95]	Leica ScanStation C10	Change detection	K-means clustering and difference analysis
[96]	Leica P40 and FARO Focus 3D	Wall segmentation	Continuous wavelet transform and dilation process
[97]	Riegl VUX-1HA and ZF Profile 9012	Displacement measurement	
[98]	Trimble TX8 and Leica C10	Defect detection	Point cloud analysis
[99]	GNSS Leica GS15 and CS15	Defect detection	Dsm analysis
[100]	Zoller + Frohlick 9012	Displacement measurement	

**Table 8:** A comprehensive summary of LiDAR-based assessment techniques for post-disaster reconnaissance.

References	Laser-Based Scanning Device	Type of Assessment	Post-Processing Method(s)
[7]	N/A	Earthquake-induced building damages	Pre- and post-Event comparison
[106]	Lecia ALS50	Collapsed building detection	Segmentation algorithm with maxent and rule-based classifiers
[107]	N/A	Earthquake-induced building damages	Difference maps
[108]	Optech LYNX Mobile Mapper M1	Hazard maps-hurricanes	Pre- and post-event comparison
[109]	Leica ScanStation C10 and ScanStation 2	Earthquake-induced building damages	N/A
[110]	Blom-CGR	Earthquake-induced building damages	Gabor Wavelets, Support Vector Machines and Random Forest
[111]	N/A	Earthquake-induced building damages	Point Cloud and BIM model comparison
[112]	Optech LYNX Mobile Mapper M1	Hurricane-induced building damages	Pre- and post-event comparison
[113]	N/A	Earthquake-induced building damages	Surface Normal Algorithms and Standard Deviation Ratio
[114]	Lecia ALS60	Earthquake-induced building damages	Classification algorithm
[115]	FARO Focus 3D	Damage evaluation	Hybrid method-3d coordinate and image-based
[116]	Leica ALS50II	Earthquake-induced building damages	Correlation coefficients of dsms
[117]	Optech LYNX Mobile Mapper M1	Structural damage evaluation	Segmentation technique
[118]	N/A	Hurricane-induced building damages	Clustering matching algorithm
[119]	Leica ALS60	Damage evaluation	Vosselman filtering method
[120]	N/A	Earthquake-induced building damages	Modal analysis

**Table 9:** A comprehensive summary of laser-based assessment techniques for other structural elements.

Reference	Laser-Based Scanning Device	Type of Assessment	Post-Processing Method(s)
[121]	Cyra Cyrax 2500 and Riegl LMS-Z210	Deflection assessment	Least-square analysis
[122]	N/A	Deflection assessment	Least-square analysis
[123]	Leica ScanStation 2	Deformation analysis	Cross-section comparison
[124]	Optech ILRIS-3D	Deformation analysis	Transformation of coordinates differences
[126]	FARO Focus 3D	Stress analysis	Polynomial surface fitting
[127]	FARO Focus 3D	Deformation analysis	Polynomial surface fitting
[128]	FARO Photon 120/20	Crack detection and characterization	Alpha-Shapes Analysis
[129]	N/A	Deformation analysis	Polynomial surface fitting
[130]	N/A	Deflection assessment	Statistical sampling technique
[131]	Leica ScanStation C10	Damage detection	Clustering algorithms (k-means, fuzzy, c-means, density-based, subtractive)
[132]	N/A	Damage detection	Differential analysis
[133]	Hexagon Absolute Arm 7325SI	Welding inspection	Photogrammetric analysis
[134]	Leica ScanStation 2	Deformation analysis	Polynomial surface fitting
[135]	Leica P20	Structural component identification	Region growing segmentation and the RANSAC
[136]	FARO Focus 3D	Damage detection	Differential analysis
[137]	N/A	Damage detection	Finite element method
[138]	Leica AT402	Deformation analysis	SfM
[139]	N/A	Structural component identification	Rbnn algorithm
[140]	Leica HDS6100	Structural component identification	Comparison of as-built and planned bim
[141]	N/A	Damage detection	Skeleton and graph-based object detection
[142]	Leica ScanStation C5	Deflection assessment	Genetic algorithm and curve fitting
[143]	Leica ScanStation 2	Deflection assessment	Interpolation analysis

## Data Availability

Not applicable.
